# Cell-Specific mRNA Therapeutics for Cardiovascular Diseases and Regeneration

**DOI:** 10.3390/jcdd11020038

**Published:** 2024-01-26

**Authors:** Raj Kishore, Ajit Magadum

**Affiliations:** Department of Cardiovascular Sciences, Temple University, Philadelphia, PA 19140, USA

**Keywords:** gene therapy, cardiovascular disease, mRNA therapeutics, modRNA, heart failure, lipid nanoparticles, exosomes, AAV vectors

## Abstract

Cardiovascular diseases (CVDs) represent a significant global health burden, demanding innovative therapeutic approaches. In recent years, mRNA therapeutics have emerged as a promising strategy to combat CVDs effectively. Unlike conventional small-molecule drugs, mRNA therapeutics enable the direct modulation of cellular functions by delivering specific mRNA molecules to target cells. This approach offers unprecedented advantages, including the ability to harness endogenous cellular machinery for protein synthesis, thus allowing precise control over gene expression without insertion into the genome. This review summarizes the current status of the potential of cell-specific mRNA therapeutics in the context of cardiovascular diseases. First, it outlines the challenges associated with traditional CVD treatments and emphasizes the need for targeted therapies. Subsequently, it elucidates the underlying principles of mRNA therapeutics and the development of advanced delivery systems to ensure cell-specificity and enhanced efficacy. Notably, innovative delivery methods such as lipid nanoparticles and exosomes have shown promise in improving the targeted delivery of mRNA to cardiac cells, activated fibroblasts, and other relevant cell types. Furthermore, the review highlights the diverse applications of cell-specific mRNA therapeutics in addressing various aspects of cardiovascular diseases, including atherosclerosis, myocardial infarction, heart failure, and arrhythmias. By modulating key regulatory genes involved in cardiomyocyte proliferation, inflammation, angiogenesis, tissue repair, and cell survival, mRNA therapeutics hold the potential to intervene at multiple stages of CVD pathogenesis. Despite its immense potential, this abstract acknowledges the challenges in translating cell-specific mRNA therapeutics from preclinical studies to clinical applications like off-target effects and delivery. In conclusion, cell-specific mRNA therapeutics have emerged as a revolutionary gene therapy approach for CVD, offering targeted interventions with the potential to significantly improve patient outcomes.

## 1. Introduction

Cardiovascular diseases (CVD) are the leading causes of death worldwide including myocardial infarction (MI), heart failure (HF), coronary artery diseases (CAD), hypertension, peripheral artery diseases (PAD), arrhythmias, cardiomyopathies and congenital heart diseases; more people die annually from CVDs than other illnesses. An estimated 19.5 million people died from CVDs in 2020, and there are expected to be over 23 million deaths from CVDs by 2030 [1]. About 5.8 million people in the United States suffer from heart failure, and nearly 700,000 new cases are diagnosed each year [1]. The global cost of CVDs was USD 863 billion in 2010. Post-MI or HF, 20–40% of cardiomyocytes (CMs) die, contributing to reduced ejection fraction and HF [2]. While it was traditionally believed that the adult mammalian heart does not regenerate, recent mouse and pig studies show regenerative potential shortly after birth, albeit lost within the first week [3,4,5]. Multiple gene expression studies in this regenerative window highlight differentially expressed genes, mainly related to mitosis and the cell cycle. Over the last two decades, various methods have induced mammalian CM proliferation, using proteins, viruses, small molecules, or transgenic models [6,7,8,9,10,11,12,13,14]. However, these approaches face limitations, such as short or long half-life, complex administration, lack of CM specificity, and potential adverse effects. There is a pressing need for novel, clinically applicable therapeutics to repair cardiac tissue injuries and reverse pathological remodeling. The standard medication for ischemic heart diseases is off-patented ACE inhibitors, vasodilators, diuretics, or water pills, which help maintain heart function for a short time [15]. Therefore, there is a need to develop novel therapeutics for this unmet need.

Gene therapy involves the delivery of genetic material into cells or tissues to treat diseases. The introduced genes can confer new functions, enhance existing ones, or replace defective variants with functional ones. This process can result in permanent changes by integrating the DNA into the cell genome or temporary alterations by maintaining the DNA in an episomal state or mRNA expression [16]. The early stages of gene therapy development faced challenges due to a limited understanding of diseases, hindering progress in the field. Advances in our knowledge of diseases are crucial for the continued improvement and success of gene therapy approaches.

In recent years, RNA has emerged as a promising therapeutic avenue for various human diseases, including CVD [17,18]. The functional roles of noncoding RNAs, encompassing long noncoding RNAs (lncRNAs), microRNAs (miRNAs), and circular RNAs (circRNAs), have come to light over the past decade, contributing to our understanding of their implications in disease processes [18]. A spectrum of synthetically synthesized RNA molecules, ranging from small interfering RNA (siRNA), short-hairpin RNA (shRNA), antisense oligonucleotides (ASOs), short activating RNA (saRNA), and RNA aptamers to single-guide RNA (sgRNA) for CRISPR/Cas9 systems, has been employed in clinical studies [19]. The success of mRNA-based vaccines against COVID-19 has propelled RNA therapeutics into the forefront of clinical development [20,21]. The distinct advantages of RNA-based therapies, including ease of dosage control, low immunogenicity, and the absence of genomic integration risks, have garnered significant attention, positioning nucleic acid-based products as a promising therapeutic approach in recent times [22].

## 2. Cardiovascular Diseases

CVDs encompass a range of disorders affecting the heart and blood vessels, representing a leading global health concern. These conditions, including coronary artery disease, heart failure, stroke, and peripheral artery disease, collectively contribute to significant morbidity and mortality worldwide. The intricate interplay of genetic, environmental, and lifestyle factors contributes to the development and progression of these diseases. Coronary artery disease (CAD), the most prevalent form of CVD, occurs when the blood vessels supplying the heart muscle become narrowed or blocked, often due to atherosclerosis [1]. This impedes blood flow, leading to angina (chest pain) or, if a vessel is completely obstructed, a heart attack. Risk factors for CAD include hypertension, high cholesterol, diabetes, smoking, and a sedentary lifestyle. Heart failure, another common CVD, results from the heart’s inability to pump blood effectively. This can arise from conditions such as CAD, hypertension, or cardiomyopathies. The compromised pumping efficiency leads to symptoms like fatigue, shortness of breath, and fluid retention. Stroke, a cerebrovascular event, occurs when blood flow to the brain is disrupted, either by a blood clot (ischemic stroke) or bleeding (hemorrhagic stroke) [1]. Hypertension, smoking, diabetes, and atrial fibrillation elevate stroke risk. Peripheral artery disease (PAD) involves the narrowing of arteries outside the heart and brain, often affecting the limbs. This can lead to pain, reduced mobility, and, in severe cases, limb-threatening ischemia.

## 3. Gene Therapy: Viral Vectors

Viruses, often associated with pathogenicity, possess an inherent ability to efficiently infiltrate host cells, exploiting the host’s gene expression machinery for replication [23]. This intrinsic capability of viruses forms the basis for the design of viral vectors, leveraging their potential to deliver and express therapeutic genes in target cells—an advantage that distinguishes them from nonviral vectors, known for their lower gene transfer efficiency. Through the replacement of the entire or a substantial portion of the viral genome with the chosen expression cassette, viral vectors can be engineered to be nonpathogenic. Viral vectors are broadly categorized into integrating and nonintegrating types [24]. In the realm of gene therapy for CVD, three prominent viral vectors take precedence: lentiviral, adenoviral, and adeno-associated virus (AAV) vectors. While lentiviral and AAV vectors hold the potential for integration into the host genome, adenoviral vector DNA remains episomal [25,26]. The initial use of lentiviral and adenoviral vectors has paved the way, but AAVs have garnered significant attention in recent years, primarily due to their notable safety advantages, marking a substantial evolution in the landscape of viral vectors for cardiovascular gene therapy [26].

## 4. Adeno-Associated Virus (AAV)

AAV, initially discovered over 40 years ago as a contaminant in a Simian adenovirus preparation, emerged as a nonpathogenic virus incapable of autonomous replication. With a diameter of approximately 25 nm, AAVs carry a single-stranded DNA genome and belong to the Parvoviridae family and Dependovirus genus, encompassing at least 13 naturally occurring serotypes and over 100 variants. Recombinant AAV (rAAV) vectors, integral to gene therapy, replace the viral genome with a selected expression cassette, flanked by viral inverted terminal repeats (ITRs). Over the past three decades, rAAVs have gained prominence in human clinical trials, notably for their safety and nonimmunogenic properties. They infect both dividing and nondividing cells, ensuring long-term gene expression. The episomal maintenance of rAAV genomes eliminates the risk of insertional mutagenesis, and sustained transgene expression has been observed for several months to years [25]. AAV’s distinct tropism, particularly AAV1, AAV6, AAV8, and AAV9, which exhibit cardiotropism, positions them as favorable vectors for cardiovascular applications [27]. However, limitations include their small insert size, slow kinetics, and modest gene expression levels. Concerns about immune responses, both preexisting and acquired, have arisen, with studies highlighting seroprevalence discrepancies among AAV serotypes [27]. Immune responses can impact transgene-expressing cells, prompting investigations into strategies to mitigate such reactions. These strategies encompass tissue-specific expression, temporary immunosuppression, capsid modification, and serotype switching. Despite these challenges, ongoing research seeks innovative solutions to enhance the clinical viability of AAV gene therapy, offering promising avenues for overcoming immune responses and advancing therapeutic applications.

## 5. Therapeutic Agents and Carriers

Therapeutic agents primarily deliver their therapeutic materials to the target location and can be administered on their own. Carriers facilitate the delivery and targeting of an effector agent. Various effector agents can be categorized into nucleotides, molecules, extracellular vesicles (EVs), cells, and tissues for therapeutic interventions. modRNA, characterized by a single-stranded structure with modified nucleotides, enables immediate and short-term expression lasting approximately two weeks, exhibiting a low immune response. The miRNA is a short, noncoding RNA, which regulates gene expression and varies widely in stability. Anti-microRNA (anti-miR) serves as an antisense inhibitor targeting specific miRNAs. DNA plasmids offer short-term expression with a moderate immune response, while adeno-associated viruses provide long-term expression with low immune reactivity. Lentiviruses, containing single-stranded RNA, also ensure long-term expression with a mild immune response. Adenoviruses, with double-stranded DNA, produce short-term expression (1–4 weeks) accompanied by a robust immune response. Small compounds, notably the tetracycline or doxycycline system, find common usage in experimental studies. Peptides or proteins, exemplified by cytokines like fibroblast growth factor and erythropoietin, undergo testing for CVD treatment. EVs, including exosomes, serve as vesicles containing therapeutic nucleic acids and/or proteins, providing short-term expression with a low immune response [28]. Cells or tissues, administered intracoronarily, carry potential risks of microvascular plugging, with most cells cleared within hours but remaining cells potentially engrafting for long-term effects. Tissues, derived from diverse stem cell types, necessitate epicardial surgical access for implantation. This comprehensive classification underscores the diverse modalities available for targeted therapeutic interventions in the realm of CVD. Various carriers play a crucial role in enhancing the delivery of effector agents. EVs, particularly exosomes, can serve as effective carriers by encapsulating viruses like adeno-associated viruses [29]. Notably, EVs offer nonimmunogenic properties, and their surface modifications or integration with bioengineered donor cells enhance their versatility [30]. Liposomes, characterized by phospholipid bilayer capsules, exhibit heterogeneity in size, limited transduction efficacy, and low target specificity. Biodegradable polymers, such as polylactic acid and poly(lactic acid-co-glycolic acid), find widespread use as carriers [28]. Hydrogels, representing hydrophilic colloidal gels, demonstrate the ability to retain viral vectors, proteins, and even cells, facilitating controlled and localized release [31]. This diverse array of carriers underscores their significance in optimizing the delivery of therapeutic agents, offering a range of options with distinct advantages and applications.

## 6. RNA Therapeutics (Nonviral Vectors)

RNAs, including protein-coding and noncoding variants like lncRNAs, miRNAs, and circRNAs, have emerged as promising therapeutics for various diseases, including CVD. Challenges like RNA stability and immunogenicity have been addressed over time, leading to recent FDA approvals for siRNA-based drugs (patisiran, givosiran) and mRNA-based COVID-19 vaccines [20,21]. The focus on mRNA, a single-stranded molecule translating genetic sequences into proteins, gained momentum after overcoming stability challenges. Pseudouridine substitution in mRNA, pioneered by Karikó et al. in 2008, enhanced translation and immune evasion. ModRNA, synthesized with 100% replacement of uridine by N1-Methylpseudouridine-5′-Triphosphate, demonstrates prompt, highly effective, dose-controlled, transient, safe, and RNase-resistant expression [22,32]. In cardiac cells, modRNA exhibits rapid translation, peak expression between 12 and 48 h, and transient kinetics. Its controlled expression and inability to integrate into the genome make modRNA a promising gene therapy platform for various diseases, including heart conditions [11,33,34,35,36,37,38,39].

Recent advancements in RNA biology, stability, and delivery systems have propelled fully synthetic mRNA-based gene delivery platforms. ModRNA’s potency, safety, and nonimmunogenicity in animal models, including large animals, make it an attractive option for regenerative therapy [35,40]. The success of mRNA-based COVID-19 vaccines in human trials further supports the potential of mRNA as a safe and effective gene therapy tool [21]. The synthetic nature and standardized manufacturing processes pave the way for large-scale production for extensive human trials and clinical applications, reducing time and costs.

## 7. Why Cell-Specific Therapeutics Are Necessary for Cardiovascular Diseases

Cell-specific gene therapy is desirable for CVD to enhance the precision and efficacy of treatment while minimizing potential off-target effects [11,34,40]. The cardiovascular system is composed of diverse cell types, including cardiomyocytes, endothelial cells, immune cells, fibroblasts, and smooth muscle cells, each with unique functions. However, achieving cell specificity is crucial to avoid unintended consequences and improve the overall safety and effectiveness of the therapy [11,34]. Here are some reasons why cell-specific gene therapy is preferred for CVD:

Precision Targeting: Different cell types within the cardiovascular system play distinct roles, and targeting a specific cell type, such as cardiomyocytes, can maximize the therapeutic impact. By using cell-specific platforms, gene therapy can be engineered to selectively express genes in the desired cell population, ensuring that the therapeutic gene is expressed where it is needed most.

Minimization of Off-Target Effects: Nonspecific transduction of cells outside the intended target can lead to off-target effects and potential adverse reactions. Cell-specific gene therapy helps minimize these off-target effects by restricting transgene expression to the specific cell type relevant to the disease pathology, reducing the risk of unintended consequences.

Enhanced Safety Profile: Achieving cell specificity contributes to the safety profile of gene therapy. By minimizing interactions with nontarget cells, the therapy is less likely to trigger immune responses or cause unintended physiological effects in unrelated tissues, contributing to the overall safety and tolerability of the treatment.

Optimized Therapeutic Outcomes: CVDs often involve specific cell populations that contribute to the disease progression or complications. Cell-specific gene therapy allows for the precise modulation of these cellular targets, potentially leading to more effective therapeutic outcomes by addressing the root causes of the disease.

In summary, cell-specific gene therapy for CVD is crucial for precision targeting, minimizing off-target effects, enhancing the safety profile, and optimizing therapeutic outcomes. This approach holds great potential for developing tailored and effective treatments for various cardiovascular conditions.

AAV9 holds substantial promise in the field of gene therapy due to its remarkable ability to effectively transduce various tissues, including the heart. Leveraging cell-specific promoters, AAV9 enables the exclusive expression of transgenes in specific cells, enhancing precision in therapeutic interventions. Despite these advantages, AAV gene therapy presents certain limitations. A notable constraint is its tendency to integrate into the chromosome, which raises concerns about potential long-term effects. Additionally, AAV has a restricted gene insertion capacity, rendering it unsuitable for expressing large genes exceeding 4.5 kilobases [16,41,42]. The duration of transgene expression in the heart is generally prolonged, often surpassing a year; however, this prolonged expression is associated with known drawbacks, including the induction of cardiac hypertrophy and arrhythmias. Moreover, a significant portion of the population, exceeding 60%, harbors neutralizing antibodies against AAVs, imposing a restriction on their widespread use in gene therapy applications [43]. These considerations underscore the importance of continued research to address these limitations and refine the application of AAV9 in gene therapy approaches.

## 8. Specific Modified mRNA Translation Systems (SMRTs)

**Figure 1:**
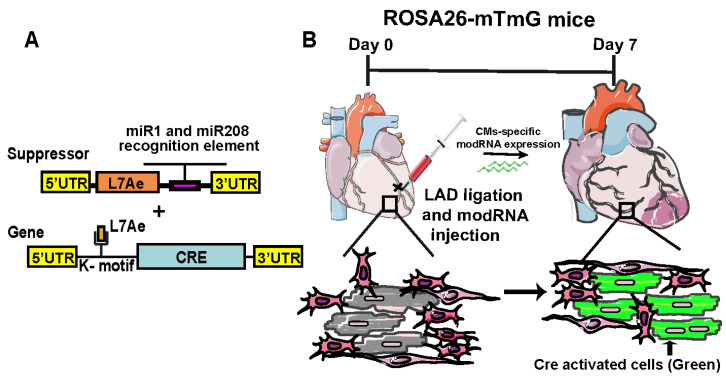
**Cardiomyocyte-specific expression of modRNA.** (**A**) CM-specific modRNA expression regulatory constructs. (**B**) Representative image of CM-specific Cre modRNA delivery and GFP expression or mT to mG switch in CMs.

Previously, there was not an mRNA-based system available to deliver any gene in a cell-specific manner in the heart. The mRNA does not differentiate the cells and they are translated by the cell’s translational machinery. There are many cellular processes in the context of the heart after injury where developing cell-specific mRNA therapeutics would be helpful; for example, the use of cell cycle inducers such as cyclins, viral proteins, and growth factors in a non-cell-specific (global) manner increases proliferation in CMs and non-CMs, which might inhibit the cardiac repair process [11,34,44]. To express any gene (modRNA) only in CMs, a CM-specific modRNA system was designed that has two distinct modRNA constructs [11]. The first modRNA construct is a suppressor or repressor modRNA (m1Ψ), which carries L7AE, an archaeal ribosomal protein that regulates the translation of a gene of interest modRNA with a kink-turn motif (k motif), a specific binding site for L7AE [45,46]. Translation of L7AE modRNA suppresses the translation of the gene-of-interest modRNA when the two are cotransfected into the cell. By adding a CMs-specific microRNA (_CMS_miR) recognition element to the L7AE gene, we can prevent L7AE translation in CMs that plentifully and mostly exclusively express the miR (“suppress the suppressor” approach), allowing the translation of the gene-of-interest modRNA precisely in CMs (Figure 1). 

Furthermore, SMRTs present a potential avenue for mitigating the impact of several detrimental cardiomyocyte-specific microRNAs following MI. Previously, it was shown that the miR recognition element could be used to decrease the number of targeted miR copies (“sponge approach”) [47,48,49]. Our strategy is not to reduce miR expression beneficial for cardiac regeneration but to reduce miR expression detrimental to cardiac repair. This approach provides a potentially complementary effect of CM proliferation to the one contributed by cell cycle inducer genes. Among these, miR208a shares the same intron as the cardiomyocyte-specific marker Myh6, inducing hypertrophy by upregulating the β-myosin heavy chain. Elevated muscle-specific miR-1 has been associated with increased cell death, while CM-specific miR-199a has been linked to impaired autophagy and the promotion of cardiac hypertrophy through mTOR activation. To investigate these effects, we designed four inactive human CD25 (ihCD25) modRNAs, each with or without recognition elements targeting these detrimental _CMS_miRs. Transfection of these modRNAs into neonatal rat heart cells followed by immunostaining for hCD25 after one day in vitro revealed distinct patterns. Similarly, in vivo delivery to adult mouse hearts four days post-MI and the collection of hearts two days later demonstrated selective translation based on recognition elements. Notably, CD25 modRNA with miR1 or miR208 recognition elements was predominantly translated in noncardiomyocytes, while CD25 modRNA with or without miR199 recognition elements showed translation in both cardiomyocytes and noncardiomyocytes, both in vitro and in vivo. These findings suggest that recognition elements for miR1 and miR208 can significantly inhibit modRNA translation in cardiomyocytes, both in vitro and in vivo [11,34]. However, it is important to clarify that our results do not necessarily imply a greater expression or functional significance of miR1/miR208 in cardiomyocytes compared to other _CMS_miRs.

To assess the immediate impact on CM-specific microRNA (_CMS_miRs)-targeted gene expression following MI, we administered modRNA containing an L7AE gene designed for cardiomyocyte-specific expression, with or without miR1/miR208 recognition elements. Two days post-administration, we harvested heart apexes to assess the expression of various miR1 and miR208 target genes. Our findings reveal that both miR1 and miR208 recognition sites function as effective sponges for their respective _CMS_miRs, leading to an upregulation of their target genes compared to no-miR modRNA [34]. Notably, modRNA carrying miR1 and miR208 (miR1-208) demonstrates a robust elevation in the expression of _CMS_miR target genes, establishing it as an optimal _CMS_miR recognition element for Synthetic messenger RNA therapeutics [34].

**Figure 2:**
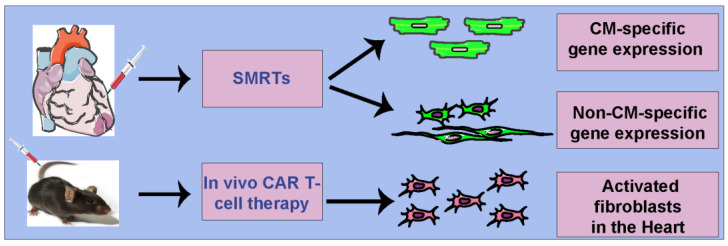
**Cell-specific modRNA delivery platforms in the heart.** SMRTs platform to target cardiomyocytes or noncardiomyocytes (direct injection in myocardium) and in vivo CAR T-cell therapy to target activated fibroblasts in CVD (intravenous injections).

To explore the impact of miR1-208 in noncardiomyocyte-specific modRNAs, we engineered nuclear green fluorescent protein (nGFP) with or without the miR1-208 recognition element for cell culture transfection, mirroring the experimental setup. Our results demonstrate that nGFP modRNA lacking _CMS_miR recognition sites is translated in both CMs and non-CMs. Conversely, nGFP modRNA equipped with miR1-208 recognition sites exhibits a significant and exclusive translation in non-CMs, sparing CMs from expression [11,34]. Similarly, the delivery of Cre recombinase modRNA with or without miR1-208 recognition leads to Cre-mediated excision of a stop codon between the two loxP sites, resulting in substantial GFP gene activation exclusively in non-CMs, not CMs, 28 days post-MI in an R26mTmG mouse MI model (Figure 1 and Figure 2) [11,34]. This substantiates our conclusion that the incorporation of the miR1-208 recognition element into the 3’-UTR of modRNA ensures translation only in non-CMs, both in vitro and in vivo.

To achieve CM specificity, we employed two distinct modRNAs. The modRNA carrying L7Ae, responsible for regulating Cre or nGFP modRNA translation through a specific L7Ae binding site (kink-turn motif), acts as a suppressor when cotransfected with either Cre or nGFP modRNA. By incorporating the miR1-208 recognition element into L7Ae modRNA, we adopted a “suppress the suppressor” approach, preventing L7Ae translation in CMs that predominantly and almost exclusively express these miRs [11,34]. This ensures that the gene-of-interest modRNA exclusively translates in CMs. Using an R26mT/mG mouse MI model, we observed that delivering Cre with the kink-turn motif (Cre K) alone activated GFP expression in both CMs and non-CMs [11]. However, simultaneous delivery of Cre K and L7Ae modRNA with miR1-208 resulted in exclusive activation of GFP expression/translation in CMs post-MI (Figure 1). In parallel experiments using our adult mouse MI model, we demonstrated that transfecting nGFP k-motif generated nGFP translation in both CMs and non-CMs. Still, cotransfection of nGFP k-motif with L7Ae modRNA carrying miR1-208 yielded sufficient nGFP for exclusive translation in CMs (Figure 1) [11]. As L7Ae is a foreign protein, it was thought that it might induce an immune response and cell death in mice. By using CD45 or TUNEL staining 7 days post-MI and modRNA with or without L7Ae in hearts, it was shown that there was no significant change between CD45-, CD3-, or TUNEL-positive cells, suggesting that L7Ae expression does not affect immune response or cell death in the heart post-MI [11].

The delivery of CM-specific Pkm2 modRNA (m1Ψ) using the SMRTs approach significantly induced the CM cell cycle 7 days post-MI without affecting the non-CMs [11]. In contrast, the global expression of Pkm2 induced both CM and non-CM cell cycles compared to the control (Luc). As discussed above, the CM-specific delivery of Pkm2 modRNA significantly improved cardiac function, cardiomyocyte proliferation, and mice survival and reduced scar size compared to the global Pkm2 modRNA post-MI [11].

The sequence alignment of miR1/miR208 in rats, mice, and humans reveals a high degree of similarity, indicating the potential applicability of SMRTs in humans [34]. Building on this, we devised two highly efficient SMRTs capable of exclusively translating mRNA in either CMs or non-CMs, both in vitro and in vivo (Figure 1 and Figure 2). Notably, SMRTs demonstrated the ability to mitigate detrimental _CMS_miRs in the heart post-MI. This innovative system offers researchers a valuable tool to assess functionality and customize gene-of-interest treatments for different cell types following MI, paving the way for more targeted and effective therapeutic interventions [34].

## 9. mRNA-Based CAR T Cell Therapy from T Cells to Target Activated Fibroblasts in CVD

Under cardiac stress induced by various insults, quiescent cardiac fibroblasts undergo activation, proliferation, and heightened secretion of matrix components [50]. Transcriptomics studies in injured mice reveal molecular distinctions between activated and homeostatic fibroblasts, with specific proteins like fibroblast activation protein (FAP) and periostin being selectively expressed by the former [51]. Genetic ablation of periostin-expressing activated fibroblasts demonstrates improved heart function in mouse models of heart fibrosis and failure [52]. Motivated by these findings, the authors proposed a novel interdisciplinary approach, merging immunotherapy and heart failure research, to develop T cell-based therapies targeting activated fibroblasts in cardiac injury. Aghajanian et al. (2019) demonstrated the effectiveness of T cells engineered with a FAP CAR in reducing fibrosis and enhancing heart function in a pressure-overload cardiac disease mouse model [53]. The ex vivo virally engineered CAR T cells from this proof-of-concept study are anticipated to persist for decades in humans.

Hypothesizing further, the authors explored the potential of a targeted lipid nanoparticle (tLNP) to deliver mRNA encoding FAP CAR directly to T cells in vivo [54]. They achieved targeted T cell delivery by attaching anti-CD5 antibodies to the LNPs, ensuring effective delivery of modRNA to T cells, resulting in FAP CAR expression and functional cytotoxic FAP CAR T cells (Figure 2) [54]. Crucially, these cells reversed both cardiac fibrosis and functional impairment in a pressure-overload injury mouse model of heart injury with a single injection of CD5-tLNP. Notably, in vivo-generated FAP CAR T cells exhibited trogocytosis, providing evidence for their functional activity (Figure 2) [54]. This integrated approach not only offers a potential therapeutic strategy for cardiac fibrosis but also demonstrates the versatility and effectiveness of RNA-LNP technology in the realm of immunotherapy.

## 10. LNPs (Lipid Nanoparticles)

LNPs, composed of lipids with hydrophilic heads and hydrophobic tails, self-assemble due to unique lipid features [55,56]. LNP-RNA systems form through hydrophobic and electrostatic interactions in an aqueous environment. LNPs are further modified with helper lipids, cholesterol, and polyethylene glycol (PEG) to enhance stability, cell entry, and circulation time [57]. Adjusting LNP components allows for tuning of their properties, with PEG content inversely affecting particle size. Studies show that particle size influences mRNA translation efficiency. PEGylation stabilizes LNPs, preventing serum protein binding and opsonization [58]. Modifying PEG acryl chain length facilitates faster shedding, reducing immune responses. Different cholesterol analogs alter LNP morphology, impacting translation efficiency and informing LNP design [58]. These modifications collectively enhance the safety, stability, and performance of LNPs for effective gene delivery [59].

Cationic lipids play a crucial role in formulating LNPs with nucleic acids, interacting with negatively charged phosphate groups for LNP integration. The use of DOTMA and DOPE lipids resulted in successful transfections in 1989, establishing the effectiveness of cationic lipids in generating LNPs [60]. Despite known cytotoxicity, commercially available Lipofectamine has been widely employed for in vitro RNA and DNA transfections. Cationic and anionic lipids, when interacting, form cone-shaped structures, promoting hexagonal HII phase formation and correlating with membrane fusion, contributing to cationic lipid toxicity [60]. Systemically delivered LNPs with a permanent surface charge interact with serum proteins, leading to rapid clearance from circulation. Cationic LNPs exhibit toxicity toward phagocytic cells in vitro and induce a robust immune response, activating interferon type I and proinflammatory cytokines [56]. While excessive immune reactions are undesirable, controlled immune response activation can be advantageous in RNA-LNP-based vaccines [61]. Despite the drawbacks of cationic lipids, their efficient nucleic acid entrapment has led to the development of pH-sensitive ionizable cationic LNPs for enhanced RNA delivery.

LNPs used in systemic nucleic acid delivery primarily incorporate ionizable cationic lipids, helper phospholipids, cholesterol, and PEG [62]. These LNPs address the toxicity of permanently cationic lipids, with patisiran being a notable FDA-approved therapeutic utilizing ionizable lipids [63]. The design of ionizable cationic lipids involves balancing their pKa value to enable RNA binding at low pH, facilitating endosomal escape while maintaining relative neutrality at physiological pH [62]. Mechanistically, cationic ionizable lipids undergo protonation in acidic endosomes, promoting non-bilayer hexagonal structures that disrupt endosomal bilayers, releasing LNP cargo into the cytoplasm. The cellular translation efficiency of LNP-RNA depends on cargo release into the cytoplasm, with varying mechanisms observed in different cell types [56]. Ethanol loading procedures have enhanced LNP production, and the development of various ionizable lipids with tailored features has expanded the versatility of LNPs. Both BioNTech and Moderna, before SARS-CoV-2 vaccines, focused on LNP-encapsulated mRNA therapeutics with properties optimized for different purposes. Research highlights the importance of tuning LNP properties for optimal expression rates in targeted tissues. Notably, ionizable lipid LNPs are the preferred carriers for clinical therapeutic RNA delivery, exemplified by anti-SARS-CoV-2 mRNA vaccines and FDA-approved treatments like patisiran for hereditary transthyretin amyloidosis [21,56,63]. As the safety and efficiency of LNP-RNA therapeutics are established, ongoing efforts aim to design cell- and organ-specific treatments for minimally invasive clinical delivery routes. Numerous trials are underway in pursuit of these objectives.

## 11. Organ-Specific LNPs

In the context of systemic delivery for protein replacement therapy, apolipoprotein E (ApoE) in blood serum has been identified to facilitate liver homing of intravenously injected lipid nanoparticles (LNPs). LNPs, particularly those with ionizable lipids, exhibit tissue tropism influenced by their charge, as observed in ApoE-dependent hepatic uptake [64]. To address excessive liver homing, a selective organ-targeting (SORT) strategy has been explored, incorporating specific lipid classes into LNPs for tissue-specific gene delivery [65]. Manipulating the charge of formulated LNPs using SORT molecules, such as DOTAP or 18PA, allows for lung- or spleen-specific gene delivery, demonstrating adaptability for therapeutic goals. Additionally, strategies for targeting specific cell subsets in the liver, such as hepatocytes and liver sinusoidal endothelial cells, have been pursued by adjusting particle size and incorporating targeting ligands [65].

Novel ionizable amino lipids and an ASSET system, utilizing cell-targeting antibodies, have been employed to enhance tissue specificity [66]. Although challenges persist in achieving systemic delivery into tumors, RNA-lipoplexes (RNA-LPX) have shown promise in cancer immunotherapy by targeting antigen-presenting cells and inducing T-cell activation. The modRNA, such as 1-methylpseudouridine (m1Ψ) RNA, contributes to reduced inflammatory responses and improved therapeutic outcomes in autoimmune disease models [22,67]. These studies underscore the importance of careful LNP design and RNA modification for tissue-specific delivery and therapeutic efficacy, providing valuable insights for advancing RNA-based therapeutics.

## 12. mRNA or ModRNA Delivery or Administration

Researchers have explored various methods for delivering RNA to cells, moving beyond the use of naked RNA, which is susceptible to RNase degradation and elicits a strong proinflammatory response [22]. Among the different formulations for RNA delivery, lipid-based LNPs have gained prominence and are the only RNA therapeutic carriers approved for clinical use. LNPs, including those used in anti-SARS-CoV-2 vaccines, have demonstrated efficacy in delivering therapeutic mRNA, with millions of doses administered globally [21,56]. While LNPs are a primary focus, alternative formulations for RNA delivery exist, such as polymers and carbohydrate polymers. Polymers like polyethylenimine (PEI) show a good affinity for nucleic acids but are limited by significant cytotoxicity, hindering their broader use in preclinical and clinical settings [56]. Polyesters represent another group of materials explored for RNA delivery, with biodegradable polyesters showing promise in lung-specific mRNA delivery for potential pulmonary disease treatment [68]. Additionally, biodegradable polymeric matrices, like LODER^TM^, have been developed for prolonged siRNA delivery in clinical trials, offering slow and sustained release in the tumor environment [69]. Carbohydrate polymers, such as chitosan, exhibit biodegradability and cationic charge for nucleic acid binding but face challenges like poor water solubility and limited target capability [56]. The diverse range of delivery systems underscores the ongoing efforts to optimize RNA delivery for therapeutic applications.

## 13. Conclusions and Perspectives

Gene therapy for CVDs has evolved significantly over the last three decades, demonstrating resilience and adaptability in the face of challenges. While no definitive breakthrough has been achieved for cardiac applications, recent developments in RNA therapeutics and gene editing technologies provide renewed optimism and a broader array of tools for addressing conditions such as MI, HF, and inherited cardiac diseases [35,40]. One major hurdle in cardiac gene therapy lies in achieving targeted and efficient delivery to the heart [35]. The specificity of therapeutic agents and their retention in the cardiac tissue are crucial factors that necessitate focused efforts. As the field progresses, an emphasis on targeted drug delivery becomes imperative. Despite challenges, gene therapy, especially with the advent of modRNA technology, highlights great promise [35].

Advancements in technology and a deeper understanding of molecular mechanisms have paved the way for more targeted and effective interventions. The utilization of modRNA technology stands out as a beacon of hope in treating CVDs, providing precise control over gene expression in a cell-specific manner [11,34]. This approach not only offers therapeutic benefits but also minimizes off-target effects, enhancing the safety profile of cardiovascular gene therapy. Delivery remains a critical challenge in mRNA therapy for cardiovascular applications. Achieving cardiac-specific delivery of therapeutic agents is crucial for efficacy and safety. Efforts to enhance cardiac specificity and retention of therapeutic agents are ongoing, and there is a growing recognition of the need for targeted drug delivery to advance the field [40]. Viruses, through evolution, have developed specific tropisms toward their cellular targets. Incorporating AAV or viral proteins into EVs holds promise for facilitating AAV or viral proteins to specific cardiac cells and overcome AAV neutralization by applying antibodies against it [70].

LNPs and other delivery platforms have played a pivotal role in overcoming challenges associated with mRNA therapy. These platforms ensure efficient and targeted delivery of therapeutic genes to the heart. LNPs, in particular, have demonstrated effectiveness in protecting mRNA molecules, facilitating cellular uptake, and enabling translation of the encoded proteins. However, it is crucial to acknowledge existing limitations, such as potential immunogenicity, off-target effects, and the need for enhanced delivery systems. Looking ahead, the future of mRNA therapy for CVD holds exciting prospects [35,37]. Refinements in delivery systems, cell-specific mRNA expression platforms, an enhanced understanding of cardiac biology, and the identification of novel therapeutic targets are expected to contribute to the development of safer and more efficacious treatments. Collaborative efforts among researchers, clinicians, and industry partners will be paramount in accelerating the translation of mRNA therapies from experimental settings to clinical practice.

One of the significant advantages of mRNA therapy lies in its ability to be tailored to individual patients. The personalized nature of this approach aligns with the broader trends in personalized medicine, heralding a new era in the management of CVDs. As researchers delve deeper into the intricacies of cardiac biology and disease mechanisms, the identification of specific mRNA targets will become more precise, allowing for even more tailored and effective treatments. Addressing challenges such as potential immunogenicity and off-target effects will be crucial for the continued success and widespread clinical application of mRNA therapy in the cardiovascular domain. Ongoing research endeavors are focused on refining delivery systems, targeted delivery, minimizing adverse effects, and optimizing the therapeutic window.

Recent studies have demonstrated that the incorporation of N1-methylpseudouridine into mRNA leads to +1 ribosomal frameshifting in vitro [71]. This phenomenon is likely a result of N1-methylpseudouridine-induced ribosome stalling during in vitro transcription (IVT) mRNA translation, particularly at ribosome slippery sequences. These frameshifting events bear significant implications for the accuracy of protein production [71]. To address the undesired consequences of +1 ribosomal frameshifting caused by N1-methylpseudouridylation, researchers are actively exploring strategies to modulate or prevent this modification. Investigation into the regulatory mechanisms, synonymous targeting of slippery sequences, and the implementation of techniques to control N1-methylpseudouridylation hold promise for developing approaches that can mitigate unintended frameshifts. Such endeavors aim to enhance the fidelity of protein translation, contributing to a more accurate and controlled cellular protein synthesis process.

In conclusion, mRNA therapy has emerged as a promising and dynamic field in the quest to address CVD. An innovative use of modRNA technology, coupled with advancements in delivery platforms, transient cell-specific delivery has opened new avenues for treating conditions that were once considered challenging. While challenges persist, the field is resilient and adaptive, continuously striving to overcome hurdles and unlock the full potential of mRNA therapy for CVD. The journey ahead involves a concerted effort from the scientific community, clinicians, and industry partners to navigate the complexities of cardiac biology and translate promising therapies into transformative clinical interventions. As mRNA therapy for CVD advances, it holds the promise of reshaping the future of cardiovascular care, offering hope to millions of individuals affected by these debilitating conditions.

## Data Availability

R.K. and A.M. filed patents in the field of modRNAs.

## Abbreviations

modRNA	modified mRNA
MI	myocardial infarction
HF	heart failure
LNP	lipid nanoparticles
miRNA	microRNA
CVD	cardiovascular diseases
CM	cardiomyocyte
CAR	chimeric antigen receptor
CRISPR	clustered regularly interspaced short palindromic repeats
FDA	Food and Drug Administration
GFP	green fluorescent protein
ARCA	anti-reverse cap analog
PEG	polyethylene glycol
Pkm2	pyruvate kinase isozyme 2
ihCD25	inactive human CD25
